# COVID-19-Associated Pulmonary Fungal Infection among Pediatric Cancer Patients, a Single Center Experience

**DOI:** 10.3390/jof8080850

**Published:** 2022-08-15

**Authors:** Youssef Madney, Lobna Shalaby, Mahmoud Hammad, Mervat Elanany, Reem Hassan, Ayda Youssef, Ibrahim Abdo, Abeer Zaki, Reham Khedr

**Affiliations:** 1 Pediatric Oncology Department, National Cancer Institute, Cairo University and Children’s Cancer Hospital Egypt (57357), Cairo 11311, Egypt; 2 Clinical Pathology Department, Faculty of Medicine, Cairo University and Children’s Cancer Hospital Egypt (57357), Cairo 11311, Egypt; 3 Clinical and Chemical Pathology Department, Faculty of Medicine, Cairo University and Children’s Cancer Hospital Egypt (57357), Cairo 11311, Egypt; 4 Radiodiagnosis Department, National Cancer Institute, Cairo University and Children’s Cancer Hospital Egypt (57357), Cairo 11311, Egypt; 5 Clinical Pharmacology Department, Children’s Cancer Hospital Egypt (57357), Cairo 11311, Egypt; 6 Clinical Research Department, Children’s Cancer Hospital Egypt (57357), Cairo 11311, Egypt

**Keywords:** COVID-associated pulmonary aspergillosis, COVID-associated mucormycosis, children, cancer

## Abstract

Patients with COVID-19 are at risk of developing secondary complications such as invasive pulmonary aspergillosis and mucormycosis. This is a retrospective study including all cancer children diagnosed with COVID-19-associated pulmonary fungal infection (CAPFI) during the period 2020–2021. A total of 200 patients were diagnosed with COVID-19, out of which 21 (10%) patients were diagnosed with CAPFI, 19 patients (90%) with COVID-aspergillosis (CAPA), and 2 (10%) patients with COVID-mucormycosis (CAM). Patients with CAPFI were classified using the “2020 ECMM/ISHAM consensus criteria”; proven in 2 (10%) patients, probable in 12 (57%), and possible in 7 (33%) patients. Although the hematological malignancy patients were already on antifungal prophylaxis, breakthrough fungal infection was reported in 16/21 (75%), 14 (65%) patients had CAPA while on echinocandin prophylaxis, while 2 (10%) patients had CAM while on voriconazole prophylaxis. Overall mortality was reported in 8 patients (38%) while CAPFI-attributable mortality was reported in 4 patients (20%). In conclusion, clinicians caring for pediatric cancer patients with COVID-19 should consider invasive pulmonary fungal infection, even if they are on antifungal prophylaxis, especially with worsening of the clinical chest condition. A better understanding of risk factors for adverse outcomes may improve clinical management in these patients.

## 1. Introduction

The severe acute respiratory syndrome coronavirus-2 (SARS-CoV-2) pandemic has markedly affected pediatric cancer patients. Children with malignancies were assumed to be at higher risk for severe coronavirus disease 2019 (COVID-19) due to their immunocompromised state; however, many studies reported that most pediatric cancer patients experience only mild to moderate COVID-19 infection [1,2,3]. Among patients with confirmed COVID-19, the frequency of cancer diagnosis has been reported to be 2% [4], with a 34% mortality risk rate [5].

Patients with primary COVID-19 have increased susceptibility to secondary pulmonary infections, and many studies have reported co-infection with *Aspergillus* spp., with an incidence ranging from 19.6% to 33.3% [6]. COVID-19-associated pulmonary aspergillosis (CAPA) is challenging to diagnose and is associated with increased mortality rate reaching up to 64.7% [6]. Early diagnosis of CAPA is crucial for successful treatment, yet conventional diagnostic tools as microscopy and culture of respiratory tract sample have only low sensitivity and specificity of around 50% [7]. Chest computed tomography (CT) may detect lung involvement at an early stage of infection [8]. COVID-19 pneumonia from invasive pulmonary aspergillosis (IPA) where diffuse bilateral lung infiltrates may obscure any diagnostic clues for invasive pulmonary aspergillosis [9]. 

The true incidence of CAPA is unknown as there is a lack of standardized diagnostic criteria with a mortality rate of about 48.4%, despite the widespread use of antifungals [10]. Data about COVID-19-associated pulmonary fungal infection (CAPFI) among pediatric cancer patients undergoing systemic chemotherapy are lacking. Those patients had other risk factors for invasive pulmonary fungal infection; whether COVID-19 infection among them will be an additional risk factor and its impact on the outcome is challenging. In this study, we aimed to describe the incidence, clinical characteristics, and outcome of CAPFI among pediatric cancer patients.

## 2. Patients and Methods 

### 2.1. Study Design 

A retrospective study included all pediatric cancer patients diagnosed with COVID-associated pulmonary fungal infections (CAPFI), either aspergillosis (CAPA) or mucormycosis (CAM) diagnosed and treated in Children Cancer Hospital Egypt (CCHE) during the period from May 2020 to December 2021. The Children’s Cancer Hospital Egypt is a tertiary pediatric oncology center in Cairo, Egypt. It is also the biggest oncology center in Africa and the Middle East, with more than 300 inpatient beds and accepting around 2000 new pediatric oncology cases annually. Data analysis included demographics, cancer diagnosis and treatment, COVID-19 severity, and management. We also analyzed the radiological, mycological, and histopathological data for COVID-associated pulmonary fungal infection. 

### 2.2. Definitions

#### 2.2.1. Definition of COVID Severity 

The severity of COVID-19 infection was classified as mild, moderate, severe, and critical [11]. The treatment protocol for COVID-19 at CCHE are shown in Table 1.

#### 2.2.2. Definition of CAPA 

A diagnosis of COVID-19-associated pulmonary aspergillosis (CAPA) was defined based on the 2020 European Confederation of Medical Mycology/International Society for Human and Animal Mycology (ECCM/ISHAM) consensus criteria [12].

Patients were classified as possible CAPA, probable CAPA, or proven CAPA:

- **Proven CAPA** is defined by invasive growth of *Aspergillus* hyphae identified by histopathology/microscopy obtained by biopsy from a pulmonary site or *Aspergillus* positive culture or positive *Aspergillus* PCR or a combination obtained from sterile aspiration.
- **Probable CAPA** requires either pulmonary nodules or cavitating infiltrate or both detected by chest CT combined with mycological evidence with at least one of the following: microscopic detection of fungal elements in bronchoalveolar lavage (BAL), or culture or serum galactomannan index > 0.5, or BAL galactomannan index ≥ 1.0.
- **Possible CAPA** requires either pulmonary nodules or cavitating infiltrate or both detected by chest CT in combination with mycological evidence obtained via non-bronchoscopic lavage.

#### 2.2.3. Definition of CAM

For diagnosis of COVID-associated mucormycosis (CAM), COVID-19 should be confirmed by a single RT PCR test, along with clinical, radiological, microbiological or histopathological evidence suggestive of mucormycosis as defined by ECMM/MSG worldwide guidance [13,14].

### 2.3. Response and Survival Outcome 

Response rate at 12-week treatment for patients using mycology study group and EORTC response criteria [15].

Definitions of response criteria are:

- Success (complete or partial response) was defined as the disappearance of all signs and symptoms of fungal infection with clinical and/or radiological improvement compatible with the responding disease.
- Failure was defined as progressive fungal infection while on therapy or death attributed to the fungal infection as a primary or contributing cause [15].

### 2.4. Statistical Analysis 

The tabulated information was presented using standard descriptive statistics. Median and interquartile range (IQR) were used to describe continuous data. One-year overall survival (OS) was estimated using the Kaplan–Meier method with survival duration calculated in days from the date of diagnostic RT-PCR. IBM-SPSS Statistics Version-20.0 was used in conducting data analyses.

## 3. Results

**Figure 1:**
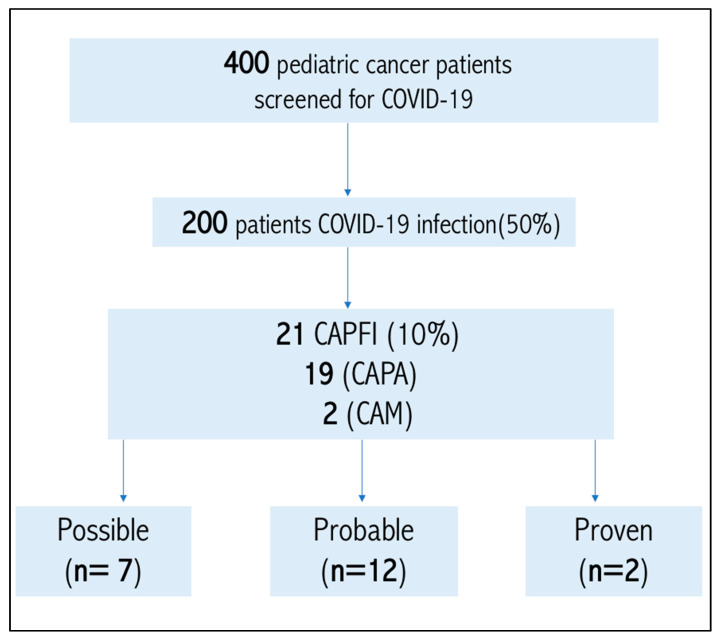
Roadmap for pediatric cancer patients diagnosed with COVID-19 associated pulmonary fungal infection among pediatric cancer patients. CAPFI: COVID-19-associated pulmonary fungal infection, CAPA: COVID-19-associated pulmonary aspergillosis, CAM: COVID-19-associated pulmonary mucormycosis.

We analyzed the electronic medical records of 400 children who presented with signs and symptoms suspicious of COVID-19; thus, PCR testing was done to prove or exclude the infection during 2020–2021. In that sample, 200/400 (50%) were COVID-19 positive, among which 21 patients had evidence of COVID-associated pulmonary fungal infection (Figure 1).

### 3.1. Clinical Features of Patients

The median age was 7 years (ranged from 2 to 21 years), and 14/21 (66%) were male. The primary diagnosis was hematological malignancy in 71% (AML = 10, ALL = 4, NHL = 1), post auto-HSCT 10% (2 patients), and solid tumor in 19% (4 patients). Most patients with hematological malignancies had CAPA during induction phase chemotherapy (52%), while three patients (14%) during consolidation phase chemotherapy, and one patient (5%) during maintenance phase treatment. The two patients of Auto-HSCT had CAPA during the pre-engraftment phase. 

The reported clinical features for developing fungal infection aside from COVID infection were prolonged severe neutropenia (ANC < 100 or more than ten days) reported in 95% (Supplementary Table S1), long-term steroid use (prednisolone dose > 0.5 mg/kg/day for >3 weeks duration) before COVID-19 diagnosis in 38% patients, and refectory/relapsing malignancy disease in 43%. All patients (100%) were on broad-spectrum antibiotics prior to COVID diagnosis. A total of 6 patients (29%) had associated bloodstream infection (BSI); 4 (19%) patients had multi-drug resistant Gram-negative bacteremia (MDR); and 2 (10%) patients had ESBL (Table 2).

### 3.2. COVID-19 Severity and Treatment

According to severity grading for COVID-19, 14 (66%) patients had moderate COVID-19, while 2 (10%) patients had a severe form, and 5 (24%) patients classified as very severe COVID-19. The median time for COVID clearance among our patients was (20 days) (range = 7–120 days).

The treatment protocol for COVID-19 in our patients includes:

- The only available antiviral in our center was Remdesivir which was given to 19 (90%) patients for 10 days during the viral phase. The immune-modulatory treatments were given for patients with lung involvement and respiratory distress: steroids were given to 21 (100%) patients, while IL-6 inhibitor (tocilizumab) was given for severe cases with respiratory distress not responding to steroid treatment for 4 (20%) patients.
- Anticoagulant LMWH prophylaxis (1 mg/kg/24 h) if moderate COVID-19 was given to 4 patients and threptic (1 mg/kg/12 h) if severe was given to 7 (52%) patients.

### 3.3. CAPA Diagnosis and Classification

A total of 21 (10%) patients were diagnosed with (CAPFI) among 200 children with cancer diagnosed with COVID-19 during the period (2020–2021). A total of 19 patients had CAPA and 2 had CAM. According to ECMM/ISHAM Consensus criteria, possible CAPA was diagnosed in 7 patients, probable in 12 patients, while 2 patients had proven CAM.

**Figure 2:**
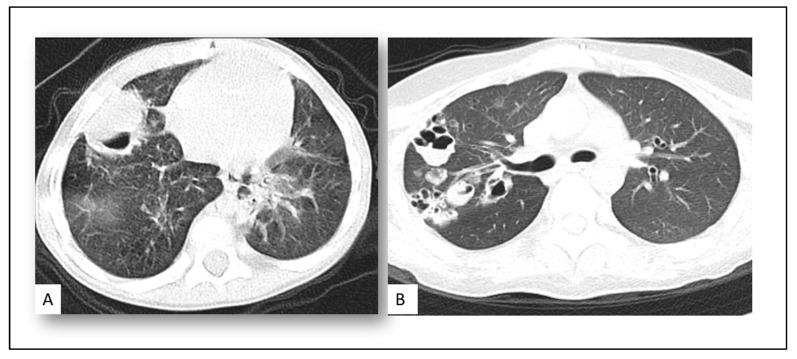
Acute myeloid leukemia patient with COVID-19-associated pulmonary aspergillosis with CT chest showed cavitary lung lesion and serum galactomannan > 1 (**A**). Patient with brain tumor with COVID-19-associated pulmonary aspergillosis with CT chest showed multiple cavitary lung lesions and BAL galactomannan > 1 (**B**).

**Figure 3:**
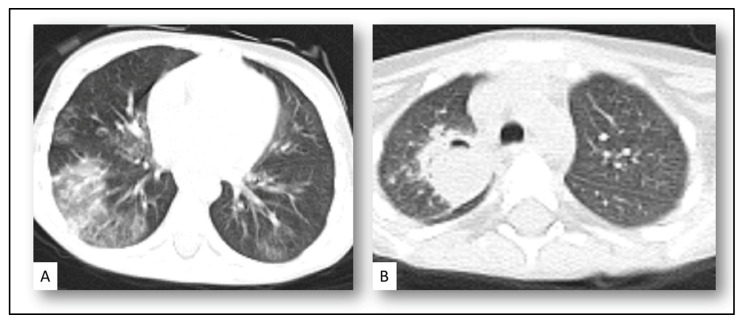
Acute myeloid leukemia patient under voriconazole prophylaxis with confirmed COVID-19 with radiological finding showed ground-glass opacities (**A**). Two weeks later patient devolved pulmonary lung lesion with crescent air sign (**B**) with voriconazole therapeutic drug level and negative galactomannan, surgical resection done with histopathology reported fungal hyphae suggesting *Mucorales* with fungal tissue PCR reported *Absidia corybifera* spp.

Diagnosis of CAPF in our patients based on clinical, radiological, microbiological, and histopathological findings (Figure 2 and Figure 3):

**Radiological finding:** The radiological finding was defined according to the fungal definition: pulmonary nodular lesion more than 1 cm with a halo sign was reported in 11 (52%) patients, wedge-shaped consolidation patch was reported in 4 (20%) patients, and 6 (28%) patients had cavitary lung lesion in CT images.

**Mycological finding:** Detection of galactomannan (GM) > 1 was reported in 13 patients (11 on serum and 2 on the BAL). Direct microscopic detection of fungal pathogens was detected in 3 patients. Tissue culture was also done on those samples and revealed *Aspergillus* flavus in one patient and *Mucorales* in the other 2 patients.

**Histopathological finding:** Only two acute myeloid leukemia patients had histopathological biopsies; the pathological finding revealed aseptate fungal spore and hyphae, branching at the right angle, with positive PAS and GMS stain suggesting *Mucorales*.

The median time from COVID-19 infection to the diagnosis of fungal infection in our study group was 11 days (range = 3–45 days, IQR 9–19 days).

### 3.4. Breakthrough Fungal Infection

Breakthrough fungal infection was reported in 16/21 (75%) patients receiving antifungal prophylaxis: 14 were on echinocandins (AML patients and ALL patients during induction), 2 patients on voriconazole (breakthrough CAM), and 2 patients on fluconazole (Hodgkin lymphoma patients undergo HSCT).

### 3.5. Treatment of CAPFI

All patients with CAPA (*n* = 19) were treated with voriconazole as first-line treatment (dose 7 mg/kg/12 h). The two patients with CAM were treated with L-ampho B (5 mg/kg for 21 days as induction followed by posaconazole syrup (200 mg oral/6 h) as maintenance treatment. All patients were monitored with drug levels to remain antifungal on the therapeutic dose. Success was reported in 13 patients (62%), with a 12-week complete response reported in 7 (33%) patients and partial response in 5 (24%) patients. Failure was reported in 8 (38%) patients (Figure S1), with 4 (20%) deaths attributable to CAPA, and the other 4 patients’ deaths were related to progressive primary malignancy (Table 3).

## 4. Discussion

In this study, we report the clinical features and diagnostic and treatment outcomes of COVID-19-associated pulmonary fungal infections (CAPFI) among pediatric cancer patients diagnosed and treated in the Children’s Cancer Hospital, Egypt. The incidence of CAPFI was 10% among patients with COVID-19, 19 patients with CAPA, and 2 with CAM. Success was reported in 62% of patients, while failure was reported in 38%. CAPA was the main attributable cause of death in 20% of patients.

Most pediatric oncology patients present with a mild to moderate course of the disease, compared to healthy children [16]. There is a risk of having a severe infection, with almost 1/10 of the patients being admitted to the ICU with a mortality rate of about 4% [17]. Age more than 11 years, hematological malignancies, and neutropenia are at higher risk for severe infections [17]. We reported 76 pediatric cancer patients with confirmed COVID-19 infection during the period between May and November 2020. Hematological malignancies constituted 87% of patients with severe to critical conditions were 35%. Most pediatric cancer patients have good clinical outcomes, with a 60-day overall survival (OS) of 86%, with mortalities occurring only among critical patients [3].

Several studies described the incidence of influenza-associated pulmonary aspergillosis (IAPA), ranging from 7% to 30% [18,19,20]. Invasive fungal infections, both *Aspergillus* and non-*Aspergillus* fungal infections have been reported in patients with severe COVID-19 pneumonia and were associated with poor outcomes [21,22]. About 5% of patients with severe COVID-19 experience lung damage due to replication of the virus, complex inflammatory process, and cytokine storm, which can lead to secondary bacterial and fungal infection after the onset of the disease [23]. The main risk factors for IFI in COVID-19 patients are neutropenia or lymphopenia, poorly controlled diabetes, antibiotic use, and immunosuppressive therapies for COVID-19, such as corticosteroids or tocilizumab [24]. In our study, neutropenia, broad-spectrum antibiotics, and exposure to steroids as immunomodulation post-COVID were the main risk factors associated with IFI. In addition, the underlying malignancy status and the prior use of steroids before COVID-19 diagnosis were reported in almost 43% of patients.

Many reports described COVID-19-associated pulmonary aspergillosis (CAPA) in critically ill patients, with incidence reported at about 20–30% [25,26]. It is still debatable whether the development of IA in these patients is directly related to COVID-19 or associated with the ARDS complication [24]. An evaluation of 186 CAPA cases showed that almost all of the cases had ARDS and were admitted to the ICU, mechanically ventilated, and nearly half of them were on corticosteroids [27]. In our study cohort, 35% of the patients were critically ill, admitted to the ICU and mechanically ventilated for ARDS. Of these patients, 20% received tocilizumab and all of them were on corticosteroids. Among patients with traditional risk factors for invasive aspergillosis such as hematological malignancies or transplant patients, COVID-19 may be an additional risk factor; however, it is unclear what factors are most important in the risk of IA [24].

Many definitions were used to classify CAPA based on clinical, radiological, and microbiological evidence combinations. We used ECCM/ISHAM definitions [12] to classify our patients; 60% were diagnosed with probable CAPA, and 10% were diagnosed as possible CAPA. The two patients with proven histopathological findings had CAM.

In our study, pulmonary nodular and cavitary lung lesions were reported at 52% and 28%, respectively. The radiological finding of CAPA is mostly nonspecific, making the diagnosis difficult. Cavitary lesions and nodules are also relatively common but may be a late finding [28].

Diagnostic tests used for diagnosis of CAPA are fungal markers, fungal PCR, cultures, and histopathology with increasing validity of diagnosis with multiple tests positivity [25,28]. Positivity in bronchoalveolar lavage fluid (BAL) is sufficient to confirm the diagnosis of CAPA; however, its use is limited during the pandemic due to the potential spread of COVID-19. Positive galactomannan antigen or PCR in blood samples is indicative of CAPA diagnosis; however, a negative test does not exclude CAPA, with improving sensitivity if other markers such as serum BDG are positive. Considering that previous antifungal therapy can impair the sensitivity of galactomannan testing [24], in our study, our diagnosis is mainly based on galactomannan testing with positivity in around 68% of our patients with limited BDG and fungal PCR availability in our center. Antifungal prophylaxis was used in 76% of our patients, which may impact the sensitivity of GM testing.

ECCM/ISHAM guidelines for treating CAPA recommend voriconazole or isavuconazole for 6–12 weeks [12]. Taking into consideration therapeutic drug monitoring and drug-drug interactions. In our study, voriconazole was the primary line treatment for patients diagnosed with CAPA, with a success rate in 62% of patients and failure in 32%. CAPA was the main attributable cause of death in 4 (20%) patients.

Due to severe lung damage caused by SARS-CoV-2, potential coinfection with less common mold infection as mucormycosis and fusarium should be considered [24]. The main risk factors for CAM are poorly controlled DM, hematological malignancies, and HSCT. CAM developed around 14 days from hospitalization and sometimes after recovery from COVID-19 with rhino-orbital, or rhino-orbital-cerebral disease, is the main clinical presentation [29]. The diagnostic approach for CAM follows the same principles in other populations; nearly all cases of CAM are classified as proven infections in contrast to CAPA [29]. High suspicion and early diagnosis should be considered for early treatment. Early surgical debridement with antifungal treatment is essential. Antifungal treatment recommendation for CAM was the same as recommended by two recent global guidelines [13,30]. In our study, two acute myeloid leukemia patients with prolonged severe neutropenia and who were on voriconazole prophylaxis had proven CAM, one patient with pulmonary disease, and the second patient had a rhino-pulmonary disease. Both patients were successfully treated with liposomal-amphotrecin-B 5 mg/kg/day and surgical debridement.

Breakthrough invasive mold infections have been reported among patients with hematological malignancies and HSCT on mold-active prophylaxis [31]. Breakthrough fungal infection was reported in most of our leukemia patients who received antifungal prophylaxis, mainly echinocandin. Breakthrough CAPA was reported in 16/21 (75%) patients receiving antifungal prophylaxis: 14 were on echinocandins (AML patients and ALL patients during induction), 2 patients were on voriconazole (breakthrough CAM), and 2 patients were on fluconazole (lymphoma patients undergoing HSCT).

Data about COVID-19 related mortality in cancer patients are controversial. In the Abi Vijenthira study, adult patients with hematologic malignancy and COVID-19 had a 34% risk of death, whereas pediatric patients had a 4% risk of death [5]. In a systematic review by Schlage and colleagues about COVID-19 in pediatric cancer comprising over 1000 patients, the attributable mortality was at least 10 times higher compared to the reports on hospitalized children without comorbidities [2]. Published data from our center regarding COVID-19 mortality was reported to be 15% while in the current study, CAPFI was associated with higher mortality rate among pediatric cancer patients as failure was reported in 38% of patients, with 20% of patients’ deaths attributable to CAPA [3].

The limitations of the study were: lack of diagnostic tools such as fungal PCR, inability to document fungal infection by BAL being highly infective or by histopathology as critically ill patients and unavailability of antifungal susceptibility testing.

In conclusion, COVID-19-associated pulmonary aspergillosis and mucormycosis are still challenging among pediatric cancer patients undergoing chemotherapy. Fungal markers are helpful in diagnosis, and rapid initiation of antifungals is mandatory. The clinician should be aware that COVID-19 is an additional risk factor for pulmonary fungal infection among pediatric cancer patients associated with a high mortality rate.

## Figures and Tables

**Table 1:** Institutional algorithm for management of pediatric cancer patients with COVID-19.

Category	Mild	Moderate	Severe	Critical
**Definition**	Patient ended chemotherapy more than 6 m - Clinically: NO respiratory distress - No evidence of pneumonia	Any pediatric cancer patient under chemotherapy with PCR confirmed test for COVID-19 with evidence of pneumonia) - No oxygen requirement - RR < 30 if more than 5 years, or <40/min if less than 5 y. - Oxygen saturation > 93% - Lung infiltration less than 50% of the lung filed.	Any pediatric cancer patient under chemotherapy with PCR confirmed test for COVID-19 with evidence of pneumonia) - RR > 30 if more than 5 years, or >40/min if less than 5 years old. - Oxygen saturation < 93% - Lung infiltration more than 50% of the lung filed.	Any pediatric cancer patient under chemotherapy with PCR confirmed test for COVID-19 with evidence of pneumonia) with: Symptoms > 1 of the following: - ARDS - Sepsis - Altered conscious level - Multiorgan failure
**Treatment**				
**Antiviral**	To be discussed for home supportive care according to every case	Remdesivir <40 kg: 5 mg/kg IV load, then 2.5 mg/Kg q24 h ≥40 Kg: 200 mg IV load, then 100 mg IV q24 h	Remdesivir	Remdesivir
**Anti-inflammatory**	No	- Methylprednisolone 1 mg/kg/day	- Methylprednisolone 1 mg/kg/12 h - +/− Tocilizumab according to clinical condition	Methylprednisolone 1 mg/kg/12 h + Tocilizumab: - <30 Kg: 12 mg/kg - ≥30 Kg: 8 mg/kg (max: 800 mg/dose)
**Anticoagulant** **Low molecular weight heparin (LMWH)**	No	- LMWH 1 mg/kg/once	- LMWH 1 mg/kg/twice per day	- LMWH 1 mg/kg/twice per day
**Antibiotics according to FN protocol**		Antibiotics according to FN protocol	Antibiotics according to FN protocol.	Antibiotics according to FN protocol

FN: Fever neutropenia, LMWH; Low molecular weight heparin.

**Table 2:** Clinical characteristics of pediatric cancer patients with COVID-19-associated pulmonary fungal infection.

Total N	N = 21
**Age range (2–21 Y), median: 7 years**	
**Sex**	
- Male	14 (66%)
- Female	7 (34%)
**Primary malignancy**	
**Hematological malignancies**	**15 (70%)**
- Acute Myeloid Leukemia (AML)	10 (48%)
- Acute Lymphoblastic Leukemia (ALL)	4 (20%)
- Non-Hodgkin Lymphoma (NHL)	1 (3%)
**Post Auto-HSCT**	**2 (10%)**
**Solid tumors**	**4 (20%)**
- Neuroblastoma	2 (10%)
- Brain tumor	2 (10%)
**Clinical features**	
- Broad spectrum antibiotics	21 (100%)
- Neutropenia	20 (90%)
- Steroid exposure prior COVID	8 (38%)
- Primary malignancy remission	
- Remission	12 (57%)
- Refractory/relapsing	9 (43%)
**Associated BSI with COVID**	**6 (30%)**
- ESBL	2 (10%)
- MDR Gram-negative bacteremia	4 (20%)
**COVID-19 Severity**	**21**
- Moderate	14 (66%)
- Severe	2 (10%)
- Very severe	5 (24%)
**COVID-19 treatment**	**21**
Antiviral	
- Remdesivir	19 (90%)
Immunomodulatory treatment	
- Steroid	21 (100%)
- Tocilizumab	4 (20%)
**CAPFI Diagnosis**	**21**
**Radiological finding**	
- Pulmonary nodule with halo sign	11 (52%)
- Pyramidal consolidation patch	4 (20%)
- Cavitary lung lesion	6 (28%)
**Mycological finding**	
- GM Serum	11 (52%)
- GM (BAL)	2 (10%)
- Tissue culture	3 (15%)
**Histopathological Finding**	2
**CAPA Definition**	**21**
- Possible	7 (33%)
- Probable	12 (57%)
- Proven	2 (10%)
**Antifungal treatment**	**21**
- Voriconazole	19 (90%)
- Liposomal ampho-B	2 (10%)
**Breakthrough CAPFI**	**16 (76%)**
- Echinocandin	14 (66%)
- Voriconazole	2 (10%)
- Fluconazole	2 (10%)
**Response to treatment**	**21**
- Successes	**13 (62%)**
- Failure	**8 (38%)**
- CAPA attributable mortality	4 (20%)

CAPFI: COVID- associated pulmonary fungal infection, ESBL: Extended spectrum Beta-Lactamase. GM: Galactomannan, BAL: Bronchoalveolar lavage, HSCT: Hematopoietic stem cell transplant, ESBL: Extended spectrum beta-lactamase, MDR: Multidrug resistance.

**Table 3:** Clinical features and treatment outcome of pediatric cancer patients with COVID-19-associated pulmonary fungal infection.

PTN	Age	Diagnosis	Disease Status	Neutropenia	Steroid	Antifungal Prophylaxis	Days from COVID-19 to Fungal	Days of COVID-19 Clearance	Fungal- Definition	COVID-19 Severity	Antifungal Treatment	Response	Survival
1	7	ALL	Relapse	Yes	Yes	Micafungin	10	-	Probable	Very Severe *	VRC	Failure	Died **
2	16	NHL	CR	Yes	Yes	No	11	120	Probable	Moderate	VRC	Success	Alive
3	9	AML	CR	Yes	No	Micafungin	3	-	Probable	Very Severe	VRC	Failure	Died **
4	15	AML	Refractory	Yes	No	Micafungin	30	6	Probable	Moderate	VRC	Success	Died
5	5	AML	CR	Yes	No	Micafungin	10	30	Probable	Moderate	VRC	Success	Died
6	2	AML	CR	Yes	No	Micafungin	21	50	Probable	Moderate	VRC	Success	Alive
7	13	AML	Refractory	Yes	No	Micafungin	21	18	Probable	Very Severe	VRC	Failure	Died **
8	21	HL	Auto-HSCT	Yes	No	Fluconazole	15	-	Probable	Moderate	VRC	Success	Alive
9	12	ALL	CR	Yes	Yes	Voriconazole	15	14	Proven	Moderate	LMB	Success	Alive
10	8	HL	Auto-HSCT	Yes	No	Fluconazole	17	21	Probable	Moderate	VRC	Success	Alive
11	2	AML	CR	Yes	Yes	Micafungin	14	7	Possible	Moderate	VRC	Success	Alive
12	5	AML	CR	Yes	No	Micafungin	10	14	Possible	Moderate	VRC	Success	Alive
13	10	AML	Refractory	Yes	No	Micafungin	10	25	Possible	Moderate	VRC	success	Died
14	3	NB	Refractory	Yes	No	No	7	7	Possible	Moderate	VRC	success	Died
15	6	BT	CR	Yes	Yes	No	10	-	Probable	Very Severe	VRC	Failure	Died **
16	4	AML	CR	Yes	No	Anidulafungin	21	10	Probable	Moderate	VRC	Success	Alive
17	6	ALL	Relapse	Yes	Yes	Micafungin	9	60	Possible	Severe	VRC	success	Alive
18	9	ALL	Relapse	Yes	Yes	Micafungin	3	14	Possible	Moderate	VRC	Success	Alive
19	3	AML	CR	Yes	No	Voriconazole	14	28	Proven	Moderate	LMB	Success	Alive
20	5	NB	Relapse	Yes	No	No	45	50	Possible	Moderate	VRC	Success	Alive
21	16	BT	Refractory	No	Yes	No	10	10	Probable	Very Severe	VRC	Failure	Died

ALL: acute lymphoblastic leukemia, AML: acute myeloid leukemia, NHL: Non-Hodgkin lymphoma, BT: brain tumor, NB: neuroblastoma, CR: complete remission, VRC: voriconazole, LMB: liposomal ampho-B. * Five patients with very severe COVID-19 were admitted to the ICU and mechanically ventilated for a duration range of 10–15 days. ** COVID-19-associated fungal infection was a direct cause of death.

## Data Availability

The data supporting this study’s findings are available from the corresponding author (Y.M.) upon reasonable request and with permission of the Children’s Cancer Hospital, Egypt.

## Supplementary Materials

The following supporting information can be downloaded at: https://www.mdpi.com/article/10.3390/jof8080850/s1, Figure S1: Overall survival (OS) for pediatric cancer patients with COVID-associated pulmonary fungal infection; Table S1: title; Correlation between mean absolute neutrophil count and COVID severity with outcome of patients with COVID-associated pulmonary fungal infection.
