# Supplementary material for: COVID-19-Associated Pulmonary Fungal Infection among Pediatric Cancer Patients, a Single Center Experience
Source: J Fungi (Basel). 2022 Aug 15;8(8):850. doi: 10.3390/jof8080850 (PMC9409978; doi:10.3390/jof8080850)
Supplement: Supplementary file 1 [file jof-08-00850-s001.zip › jof-1838552-supplementary.pdf]

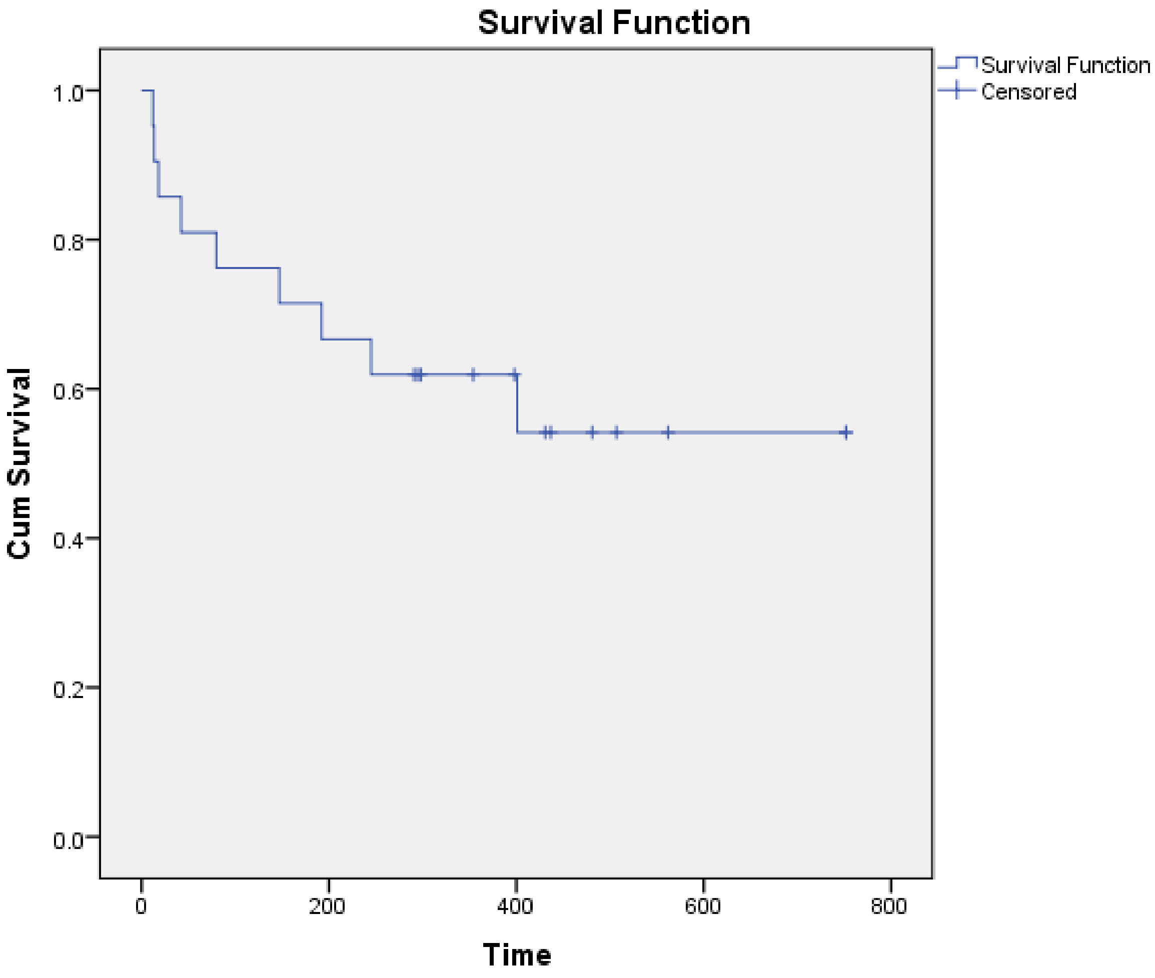

Supplementary Figure S1: Overall survival (OS) for pediatric cancer patients with COVID-associated pulmonary fungal infection.

Supplementary Table S1: correlation between mean absolute neutrophil count and COVID severity with outcome of patients with COVID- associated pulmonary fungal infection.

Mean ANC	Category	Duration of neutropenia/day	COVID-severity	Survival
10	<500	>10 days	Very severe	Died *
1900	>500	<10 days	Moderate	Alive
218	<500	>10 days	Very severe	Died *
24	<500	>10 days	Moderate	Died
224	<500	<10 days	Moderate	Died
112	<500	>10 days	Severe	Alive
69	<500	>10 days	Very severe	Died *
1000	>500	<10 days	Moderate	Alive
170	<500	<10 days	Moderate	Alive
425	<500	<10 days	Moderate	Alive
98	<500	<10 days	Moderate	Alive
103	<500	<10 days	Moderate	Alive
22	<500	>10 days	Moderate	Died
300	<500	<10 days	Moderate	Died
6600	>500	<10 days	Very severe	Died*
324	<500	<10 days	Moderate	Alive
500	>500	<10 days	Severe	Alive
185	<500	<10 days	Moderate	Alive
286	<500	<10 days	Moderate	Alive
436	<500	<10 days	Moderate	Alive
8200	>500	<10 days	Very severe	Died

ANC: Absolute neutrophil count. * COVID-associated fungal infection was a direct cause of death.

- 16/21 (76%) had a mean neutrophilic value of less than 500 (6 patients with a duration of more than 10 days while 10 patients had a duration less than 10 days).
- Of 8 patients who died, 6 patients had a mean neutrophilic value of less than 500.
- Of 4 patients with CCOVID-19-associated pulmonary fungal infection (CAPFI) were an attributable cause of death, 3 patients had a mean neutrophilic value of less than 500 with a more than 10 days duration.
- When stratified according to severity of COVID, there is statistically significant association between severe COVID- 19 disease and mortality.
